# Impact of grazing dairy steers on winter rye (*Secale cereale*) versus winter wheat (*Triticum aestivum*) and effects on meat quality, fatty acid and amino acid profiles, and consumer acceptability of organic beef

**DOI:** 10.1371/journal.pone.0187686

**Published:** 2017-11-03

**Authors:** Hannah N. Phillips, Bradley J. Heins, Kathleen Delate, Robert Turnbull

**Affiliations:** 1 West Central Research and Outreach Center, University of Minnesota, Morris, Minnesota, United States of America; 2 Department of Animal Science, University of Minnesota, St. Paul, Minnesota, United States of America; 3 Departments of Agronomy and Horticulture, Iowa State University, Ames, Iowa, United States of America; University of Illinois, UNITED STATES

## Abstract

Meat from Holstein and crossbred organic dairy steers finished on winter rye and winter wheat pastures was evaluated and compared for meat quality, fatty acid and amino acid profiles, and consumer acceptability. Two adjacent 4-ha plots were established with winter rye or winter wheat cover crops in September 2015 at the University of Minnesota West Central Research and Outreach Center (Morris, MN). During spring of 2015, 30 steers were assigned to one of three replicate breed groups at birth. Breed groups were comprised of: Holstein (**HOL**; n = 10), crossbreds comprised of Montbéliarde, Viking Red, and HOL (**MVH**; n = 10), and crossbreds comprised of Normande, Jersey, and Viking Red (**NJV**; n = 10). Dairy steers were maintained in their respective replicate breed group from three days of age until harvest. After weaning, steers were fed an organic total mixed ration of organic corn silage, alfalfa silage, corn, soybean meal, and minerals until spring 2016. Breed groups were randomly assigned to winter rye or winter wheat and rotationally grazed from spring until early summer of 2016. For statistical analysis, independent variables were fixed effects of breed, forage, and the interaction of breed and forage, with replicated group as a random effect. Specific contrast statements were used to compare HOL versus crossbred steers. Fat from crossbreds had 13% greater omega-3 fatty acids than HOL steers. Furthermore, the omega-6/3 ratio was 14% lower in fat from crossbreds than HOL steers. For consumer acceptability, steaks from steers grazed on winter wheat had greater overall liking than steers grazed on winter rye. Steak from crossbreeds had greater overall liking than HOL steers. The results suggest improvement in fatty acids and sensory attributes of beef from crossbred dairy steers compared to HOL steers, as well as those finished on winter wheat compared to winter rye.

## Introduction

The organic beef industry is still developing and accounts for a small, but growing, part in total organic sales. Organic beef is the fastest growing segment in the organic industry and increased by 46% between 1997 and 2007. Furthermore, organic beef herds were on a steady increase between 2000 and 2005 [1]. According to the USDA-National Organic Program (**NOP**) [2], cattle must consume at least 30% of their daily dry matter intake from pasture during the grazing season, except during the finishing phase, which must not exceed one-fifth of the animal’s life (up to 120 days). However, there is a high consumer preference for “grass-fed” or forage-finished beef in the United States, which is perceived as more healthy and as having less impact on the environment compared to grain-finished beef [3]. Because of the growing trend in the organic and forage-finished beef market, cattle producers may capitalize on forage for grazing and organic dairy bull calves may represent a potential new resource for organic forage-finished beef in the United States.

Beef may be a contributing source of unhealthy fats in human diets, like some saturated fatty acids (**SFAs**) and *trans* fats, which are main health concerns among consumers [4,5]. However, beef also contains many beneficial fatty acids (**FAs**), such as omega-3 (**n-3**) (especially docosahexaenoic [C22:6n-3], eicosapentaenoic [C20:5n-3], and α-linolenic [C18:3n-3] acids) and long-chain *cis*-polyunsaturated fatty acids (**PUFAs**) [4,6]. These beneficial FAs have been studied extensively in human diets and play important roles in cardiovascular, cognitive, and inflammatory functions [5]. Forage-finished beef contains greater n-3 and PUFAs, and a lower omega-6/3 (**n-6/3**) ratio compared to grain-finished beef [6–13]. Furthermore, all nine essential amino acids (**AAs**) important to the human diet are in beef and a greater concentration of essential AAs are found in forage-finished beef compared to grain-finished beef [14]. Beneficial FAs and AAs in organic and forage-finished beef may influence consumer preference [4,6,15]; however, some consumers prefer conventionally raised beef over organic and forage-finished beef due to differences in flavor and palatability sensory attributes [6,16].

According to the USDA-NOP [2], all organic farms must maintain an active soil building plan. As more concern is placed on soil health, the emphasis on soil erosion and nutrient leaching have become the main reasons to utilize winter cover crops in rotation with other crops [17]. In the Upper Midwest, winter cover crops may be planted in the fall and grazed early next spring to extend the grazing season for livestock. Cover crops may be a useful strategy because one of the main obstacles that organic and forage-finished beef producers face is lack of supply of high quality forages for pasture-based feed [18]. Extending the grazing season not only reduces the need to store feed, but the FA profile in muscle and adipose tissue improves as the grazing duration increases [19]. Increasing the grazing duration with cover crops may help producers provide pasture-based feed, improve FA profiles of beef in terms of human health, and meet the demands for forage-finished beef.

As the organic forage-finished beef industry continues to grow, it is important to understand factors that affect meat quality, characteristics of beef that influence human health, and sensory attributes of cooked beef. Research on alternative breeds and forage types that influence meat quality, FA and AA profiles, and sensory attributes in an organic forage-finished production system is lacking. Therefore, the objectives of this study were to compare beef from Holstein and crossbred dairy steers grazed and finished on winter rye (*Secale cereale*; **WR**) and winter wheat (*Triticum aestivum*; **WW**) for meat quality characteristics, FA and AA profiles, and consumer acceptability.

## Materials and methods

### Ethical statement

The current study was conducted at the University of Minnesota West Central Research and Outreach Center (**WCROC**) organic dairy in Morris, Minnesota. All animal care and management for this specific study was approved by the University of Minnesota Institutional Animal Care and Use Committee (Animal Subjects Code number 1411-32060A). The University of Minnesota’s Institutional Review Board approved all recruiting and experimental procedures. with human subjects for the consumer panel evaluation of sensory attributes (Number 0906E67481). Participants in the consumer panel provided written informed consent to participate in the study. The research dairy at the WCROC has a 300-head low-input and organic grazing system. Furthermore, the organic dairy has maintained organic certification since June 2010. The pastures in the current study were not irrigated and no soil amendments were applied.

### Experimental approach

Thirty bull calves were born at the WCROC from March to May 2015 and assigned to one of three replicated breed groups at birth. Breed groups were (1) purebred Holstein (**HOL**, n = 10), (2) crossbreds comprised of Montbéliarde, Viking Red, and Holstein (**MVH**, n = 10), and (3) crossbreds comprised of Normande, Jersey, and Viking Red (**NJV**, n = 10). The Viking Red breed was formed by combining the genetic improvement programs for the Swedish Red, Finnish Ayrshire, and Danish Red breeds, which have historically shared ancestry and similar selection criteria. Bull calves were separated at birth from their dams, housed indoors in individual pens, castrated, and fed 2 L of colostrum per 41 kg of body weight twice daily for three days. After three days of age, calves were group housed in large hutches bedded with organic wheat straw. A total of six groups of five calves were established (n = 30). Calves were fed 6 L of unpasteurized, organic milk once daily using a 10-calf Skellerup peach teat feeder (Skellerup Industries, Christchurch, New Zealand) which was washed and disinfected between each feeding. At four days of age, calves were offered starter grain *ad libitum* and were weaned when calves consumed 0.91 kg of starter grain per day at an average age of 10 weeks of age. After weaning, steers were relocated to a loose confinement barn, remained in their respective groups, and were fed an organic total mixed ration diet consisting of organic corn silage, alfalfa silage, corn, soybean meal, and minerals from the time of weaning until 25 April 2016. One NJV steer was removed from the study one month prior to grazing due to death from peritonitis, which was diagnosed by a veterinarian.

During spring of 2016, dairy steers grazed either WR (n = 15) or WW (n = 14) cover crops in the vegetative state. The WR and WW were planted on 10 September 2015 on two adjacent 4-ha plots. On 25 April 2016, each replicate breed group was randomly assigned to either WR or WW and rotationally grazed until 13 June 2016 for 7 weeks with supplemented free-choice certified organic minerals. The WR and WW cover crops were balanced for steer breed. Briefly, for forage quality of grazed cover crops, the dry matter was lower (*P* < 0.05) for WR (21.2%) compared to WW (23.6%). Crude protein was 17.6% and 19.0% for WR and WW, respectively (*P* < 0.05). Total tract neutral detergent fiber digestibility, used to measure the energy of forages, was 56.2% and 55.5% for WR and WW, respectively (*P* = 0.61).

#### Carcass measurements

The dairy steers were sent for harvest and meat fabrication on two separate dates at a commercial abattoir approved for organic harvest (Lorentz Meats, Organic Prairie, Cannon Falls, MN). The first group of HOL, MVH, and NJV steers were harvested on 27 July 2016 and the second group of HOL, MVH, and NJV steers were harvested on 21 September 2016. The steers were harvested at lower carcass weights because of lower marketability of large organic carcasses at high prices. The organic market values carcasses at a smaller weight than the conventional beef market.

Live body weight was recorded immediately prior to harvest and hot carcass weight was recorded immediately after harvest. Postharvest carcasses were chilled for 24 hours at 4°C according to North American Meat Processors [20] guidelines, and back fat thickness, ribeye area, percentage of kidney, pelvic, and heart fat, marbling, maturity, quality grade, and yield grade were recorded for each carcass.

#### Strip loin collection

Each carcass was fabricated according to North American Meat Processors [20] guidelines. One strip loin (*longissimus dorsi*) was removed from each carcass. Strip loins were identified using carcass identification tags during harvest and were followed through fabrication and vacuum-packaging.

Strip loins were maintained at 2°C during transportation to the University of Minnesota WCROC in Morris, MN where they were aged for 10 days at 2°C. After aging, strip loins were frozen at -20°C until further evaluation of meat quality and consumer sensory attributes. During November 2016, six 2.54-cm thick, frozen steaks were cut from the cranial end of each strip loin at the University of Minnesota Meat Laboratory (St. Paul, MN). The most cranial steak of the six steaks cut from the frozen strip loin was used for Warner-Bratzler shear force (**WBSF**) analysis. The next two cranial steaks were used for the objective color score analysis, and the remaining three steaks were used for the consumer sensory panel.

#### Toughness determination and objective color score

Toughness was measured on one steak from each strip loin using the WBSF instrument (G-R Elec. Mfg. Co., Manhattan, KS) at the University of Minnesota Meat Laboratory. Vacuum-sealed steaks were removed from the freezer and thawed for 24 hours at 4°C, unpackaged, wrapped in aluminum foil, and cooked in an electric oven to a final internal temperature of 71°C. Each steak was cooled to 4°C for 24 hours, then warmed to room temperature for two hours. Six 1.27-cm cores were removed from each steak parallel to the muscle fiber orientation using a hand-coring device. The average of the six cores from each steak was used as a single peak shear force measurement for each steer.

The color of each steak was measured using a HunterLab Miniscan XE Plus spectrophotometer equipped with a 6-mm aperture (HunterLab Associates Inc., Reston, VA). Objective color score values were L* (brightness, 0 = black and 100 = white), a* (redness/greenness, positive values = red and negative values = green), and b* (yellowness/blueness, positive values = yellow and negative values = blue), following procedures established by the Commission International de l’Éclairage [21]. Two vacuum-sealed, frozen steaks (two replicates) from each steer were thawed for 24 hours at 4°C, unpackaged, and exposed to the air in 4°C for two hours before measuring color scores. Readings for each of the L*, a*, and b* values were taken at three random locations on the surface of the steak exposed to the light. Readings were averaged for each steak at the time of evaluation.

#### Fatty acid profiles

Back fat samples (approximately 6.4 x 0.5-cm) were collected from all carcasses 72 hours postharvest at the abattoir. Samples were placed in Whirl-Pak^®^ bags (Nasco, Fort Atkinson, WI), transported on ice at 2°C to the University of Minnesota WCROC, and shipped on ice at 2°C in a polystyrene insulated container overnight to Minnesota Valley Testing Laboratories (New Ulm, MN) for FA profile analyses.

The FAs were determined according to AOAC method 996.06 [22] by using gas chromatography. Lipids were extracted from a 100 to 200 mg sample of finely ground fat. Pyrogallic acid was added to reduce oxidation of FAs during the analysis. The triglyceride, triundecanoin (C11:0), was added as an internal standard. Lipids were extracted in ether and then methylated to fatty acid methyl esters using Bromine trifluoride in methanol. The fatty acid methyl esters were quantitatively measured by capillary gas chromatography against the triundecanoin standard. Total fat was calculated as the sum of individual FAs expressed as triglyceride equivalents, and saturated and unsaturated fats were calculated as the sum of their respective FAs. Individual FAs are reported in percent weight of the total fat. The n-3 FA is reported as the sum of: α-linolenic (C18:3n-3), eicosatrienoic (C20:3n-3), eicosapentaenoic (C20:5n-3), and docosahexaenoic (C22:6n-3) individual PUFAs. The omega-6 (**n-6**) FA is reported as the sum of linoleic (C18:2n-6), γ-linolenic (C18:3n-6), eicosadienoic (C20:2n-6), arachidonic (C20:4n-6), docosadienoic (C22:2n-6), and docosatetraenoic (C22:4n-6) individual PUFAs.

#### Amino acid profiles

Meat samples (approximately 6.4 x 0.5-cm) were collected from all carcasses 72 hours postharvest at the abattoir from the strip loin. Samples were placed Whirl-Pak^®^ bags, transported on ice at 2°C to the University of Minnesota WCROC. Samples were aged for 10 days at 2°C. After aging, samples were shipped on ice at 2°C in a polystyrene insulated container overnight to Minnesota Valley Testing Laboratories for AA profile analyses using high performance liquid chromatography.

The AAs were determined according to AOAC method 994.12 [23] by extracting AAs from a sample equivalent to 20 mg of protein. Cysteine, methionine, and taurine were quantified from the performic acid oxidation with acid hydrolysis extraction. The remaining AAs were quantified from the acid hydrolysis extraction. Total protein is reported in percent weight of sample and individual AAs are reported in percent weight of total protein.

#### Consumer sensory evaluation

The University of Minnesota Institutional Review Board approved recruiting and experimental procedures with human subjects for the beef consumer panel evaluation of sensory attributes. The University of Minnesota’s Food Science and Nutrition Sensory Center (St. Paul, MN) recruited 108 consumers. Consumers were at least 18 years or older, had no food allergies, and had consumed beef within the past month. All consumers were paid $5 for participation in the sensory panel.

Steaks were thawed for 72 hours at 4°C in vacuum-sealed packages then unpackaged. Individual steaks were wrapped in aluminum foil, baked to an internal temperature of 71°C, and cut into 1-cm cubes. Each panelist received two pieces of steak per steer group in lidded 30 mL plastic soufflé cups coded with random three-digit codes. To maintain sample-serving temperature, cups were nested in insulated foam trays. Beef from the six steer groups was served to panelists in two sets of three samples on one tray. The first set corresponded to steers grazed on WW, and the second set corresponded to steers grazed on WR. The three breed samples within each set were balanced for order and carryover effects by personnel from the University of Minnesota Sensory Center using a Latin square design with SIMS Sensory Evaluation Testing Software (http://www.sims2000.com/). Consumers were instructed to consume the first cube and rate it for overall liking, liking of flavor, and liking of texture. Panelists were then instructed to consume the second cube and rate the intensity of toughness, juiciness, and off-flavor. Liking ratings were made on 120-point labeled affective magnitude scales (0 = greatest imaginable disliking and 120 = greatest imaginable liking), with the left-most end labeled *strongest dislike imaginable* and the right-most end labeled *strongest like imaginable*. Intensity ratings were made on 20-point line scales (0 = none and 20 = extremely tough, extremely juicy, and extremely intense, respectively) with the left-most ends labeled *none* and the right-most ends labeled *extremely tough*, *extremely juicy*, and *extremely intense*, respectively. Panelists repeated this process for each of the six steer groups.

### Statistical analysis

For statistical analysis of carcass measurements, the independent variables were fixed effects of forage and breed, with group nested within the forage and breed interaction as a random effect. Each carcass measurement was averaged for each steer group and the average was used as a single measurement for each group. For statistical analysis of WBSF, objective color score, FAs, and AAs, independent variables were fixed effects of breed, forage, and the interaction of breed and forage, with replicated group as a random effect. Replication number was included in the model for analysis of objective color score as a random effect. For the consumer sensory evaluation and analysis of like/dislike categories, independent variables were fixed effects of breed, forage, and the interaction of breed and forage, with consumer as a random effect. The chi-square test of SAS [24] was used to obtain percentages for like/dislike categories for the sensory evaluation. The MIXED procedure of SAS [24] was used to obtain least squares means and solutions for all analyses, and conduct the analysis of variance. Furthermore, specific contrast statements were used to compare HOL steers versus crossbred (MVH and NJV) steers.

Furthermore, principal component analysis [25] and correlations were conducted between select carcass variables, as well as sensory attributes with SAS software [24]. Overall liking, flavor, flavor, texture, toughness, juiciness, and off-flavor from the consumer panel where utilized to study the relationships with WBSF and objective color scores.

## Results and discussion

### Carcass quality measurements

Least squares means and standard errors for carcass measurements, WBSF, and objective color scores are in Table 1. All steers had a kidney, pelvic, and heart fat percentage of 1.0 and a maturity grading of A (not included in Table 1). The age at harvest (not included in Table 1) was not different (*P* > 0.10) for steers grazed on WR (487 ± 10.3 d) and WW (495 ± 10.3 d), as well as for HOL (492 ± 12.6 d), MVH (485 ± 12.6 d), and NJV (497 ± 12.6 d) steers.

**Table 1:** Least squares means and standard errors of means for carcass quality measurements, WBSF, and objective color scores for steers grazed on winter rye and winter wheat and for HOL, MVH crossbred, and NJV crossbred dairy steers.

Measurement	Cover crop				Breed group^1^						HOL vs crossbred
	Winter rye		Winter wheat		HOL		MVH		NJV		
	Mean	SE	Mean	SE	Mean	SE	Mean	SE	Mean	SE	*P-value*
Harvest weight, kg	470.2	3.5	471.1	3.5	484.3^a^	4.3	492.2^a^	4.3	435.5^b^	4.3	NS
Hot carcass weight, kg	225.0	4.4	230.4	4.4	231.8^A^^B^	5.4	239.9^A^	5.4	211.4^B^	5.4	NS
Dressing, %	47.8	0.94	49.0	0.94	47.9	1.1	48.8	1.1	48.5	1.1	NS
Marbling score^2^	1.9	0.16	2.1	0.16	1.9	0.20	2.1	0.20	2.0	0.20	NS
Back fat, cm	0.27	0.04	0.30	0.04	0.25	0.05	0.28	0.05	0.32	0.05	NS
Ribeye area, cm^2^	50.3	3.1	48.2	3.1	47.3	3.8	52.7	3.8	47.7	3.8	NS
Yield grade	1.9	0.09	1.9	0.09	1.9	0.11	1.9	0.11	1.9	0.11	NS
Quality grade of select and greater, %	66.7	17.0	80.0	17.0	70.0	20.8	80.0	20.8	70.0	20.8	NS
WBSF^3^, kg	3.9^A^	0.32	3.0^B^	0.33	3.9	0.39	3.6	0.39	2.9	0.42	NS
L*^4^	28.2	0.37	27.6	0.38	29.0^a^	0.45	26.6^b^	0.45	28.2^a^	0.47	0.01
a*^4^	12.5	0.33	12.0	0.34	12.1	0.40	12.2	0.40	12.5	0.43	NS
b*^4^	10.3	0.27	10.0	0.28	10.3	0.33	10.0	0.33	10.1	0.35	NS

^a,b^ Means within a row for cover crops or dairy steers without common superscript letters are different at *P* < 0.05.

^A,B^ Means within a row for cover crops or dairy steers without common superscript letters are different at *P* < 0.10.

^1^ HOL = Holstein; MVH = crossbreed comprised of Montbéliarde, Viking red, and Holstein; NJV = crossbreed comprised of Normande, Jersey, and Viking Red.

^2^ Slightly abundant = 5; moderate = 4; small = 3; slight = 2; traces = 1.

^3^ Warner-Bratzler shear force

^4^ L* = brightness (0 = black; 100 = white); a* = redness/greenness (positive values = red; negative values = green); b* = yellowness/blueness (positive values = yellow; negative values = blue).

Steers grazed on WR (470.2 kg) and WW (471.1 kg) had similar (*P* > 0.10) harvest weights. Furthermore, carcasses from steers grazed on WR (225.0 kg and 47.8%) and WW (230.4 kg and 49.0%) had similar hot carcass weight and dressing percent, respectively. For the grade of intermuscular fat, the marbling score of carcasses was similar (*P* > 0.10) for steers grazed on WR (1.9) and WW (2.1). These results are similar to those found in another study [26] comparing carcasses from steers grazed on ryegrass and ryegrass/chicory mixture pastures. Their results reported similar harvest weights, hot carcass weights, dressing percentages, and marbling scores between steers grazed on different pasture species. Furthermore, the back fat thickness, ribeye area, yield grade, and percent of carcasses with a quality grade of select or greater was similar (*P* > 0.10) for carcasses from steers grazed on WR and WW.

For steer breed groups, the HOL (484.3 kg) and MVH (492.2 kg) steers had greater (*P* < 0.05) harvest weights than the NJV (435.5 kg) steers. Carcasses from MVH (239.9 kg) steers tended to have a greater (*P* < 0.10) hot carcass weight than carcasses from NJV (211.4 kg) steers; however, hot carcass weight from HOL (231.8 kg) steers were similar (*P* > 0.10) to MVH and NJV steers. These results are similar to those found in another study [27], which reported that HOL steers had a heavier live weight and hot carcass weight than Jersey x HOL crossbred steers. Furthermore, the HOL (47.9%), MVH (48.8%), and NJV (48.5%) carcasses had similar (*P* > 0.10) dressing percentages. For the grade of intermuscular fat, the marbling scores of carcasses were similar (*P* > 0.10) between HOL (1.9), MVH (2.1), and NJV (2.0) steers. Findings in McNamee et al. [28] reported a lower marbling score for Jersey x HOL crossbred carcasses compared to HOL carcasses; however, the Normande and Viking Red genetics in the NJV crossbreed may have played a role in marbling score similarities between HOL and NJV breeds in the current study. Furthermore, the back fat thickness, ribeye area, yield grade, and percent of carcasses with a quality grade of select or greater were similar (*P* > 0.10) for carcasses from HOL, MVH, and NJV steers. Carcass quality measurements were comparable to what was reported by Bjorklund et al. [29] for organic grass-fed dairy steers from similar genetics. The results of the current study report similarities with research conducted with young beef bulls. Cuvelier et al. [30] reported Angus young bulls had lower carcass weights and lower muscling compared with Limousin and Belgian Blue young bulls. The study concluded that producers may select alternative breeds of cattle depending on economic conditions. Therefore, based on the results of this study and other studies [27,28,30] breed of cattle (dairy, beef, or crossbred) may have influence that on carcass characteristics, as well as meat quality.

#### Shear force of steaks

For the WBSF (Table 1) of cooked steaks, the steers grazed on WR (3.9 kg) tended to have a greater (*P* < 0.10) WBSF than steers grazed on WW (3.0 kg). Similar to the current study, Duckett et al. [31] reported steers which grazed forage species of mixed pasture, alfalfa, or pearl millet did not influence the WBSF of steaks.

For steer breed groups, steaks from HOL (3.9 kg), MVH (3.6 kg), and NJV (2.9 kg) steers had similar (*P* > 0.10) WBSF. The WBSF values for steaks in the current study are higher than reported by Bjorklund et al. [6] from steers of similar genetics, indicating that the beef in the current study may be more tender based on the WR and WW grazing conditions. The results from the WBSF test are similar to results found by McNamee et al. [28] who reported similar WBSF values for steaks from HOL, Norwegian Red x HOL, and Jersey x HOL steers. Findings in the current study are different than those found by Christensen et al. [32], which found similar WBSF for steaks from Danish Red (similar to Viking Red) and HOL steers; however, the study also reported that steaks from Jersey steers had greater WBSF than steaks from HOL steers. The NJV dairy steer breed was also comprised of Normande genetics, which may have played a role in similar WBSF values between breeds in the current study. For young beef bulls, WBSF was not different between Angus, Belgian Blue, and Limousin young bulls [30].

The current study only used one cut of meat to study the differences in toughness and tenderness. Recently, Bonny et al. [33], reported that differences in meat quality depends on the cut of beef, and breed also may influence eating quality of beef. The tenderloin (*M*. *psoas major*) had the highest meat quality score for beef, crossbreds, and dairy breeds. The strip loin, which was utilized in the current study, was rated fourth in meat quality scores out of 16 cuts of dairy beef. Quite possibly, results for WBSF among breeds or forage species may have been different if an alternative cut of beef was utilized in the study.

#### Objective color score of steaks

For objective color scores (Table 1), no differences (*P* > 0.10) were found between steaks from steers grazed on WR and WW for L*, a*, and b*. These results are similar to those found in another study [31], which reported similar L*, a*, and b* values for steaks from steers grazed on mixed pasture, alfalfa, and pearl millet.

For steer breed groups, steaks from HOL (29.0) and NJV (28.2) steers had greater (*P* < 0.05) L* values than MVH (26.6) steers, and steaks from crossbred steers had a lower (*P* = 0.01) L* value than HOL steers. However, steaks from HOL, MVH, and NJV steers had similar a* and b* values. Results from another study [28] reported similar L*, a*, and b* values between HOL, Norwegian Red x HOL, and Jersey x HOL steaks; however, the genetics of crossbred steers in the current study may have played a role in the darker color of steaks compared to HOL steers. Cuvelier et al. [30] reported differences in L* and a* values depending on the breed of young beef bulls, and Angus bulls had lower L* and higher a* compared with Belgian Blue bulls.

### Fatty acid profiles

Least squares means and standard errors for FAs of back fat from steers grazed on WR and WW are in Table 2, and the least squares means and standard errors for FAs of back fat from HOL, MVH, and NJV steers are in Table 3. Fatty acids from steers grazed on WR and WW, and from HOL, MVH, and NJV steers had the same values (< 0.10% weight of total fat) for caproic (C6:0), caprylic (C8:0), capric (C10:0), lauric (C12:0), tridecanoic (C13:0), behenic (C22:0), erucic (C22:1), lignoceric (C24:0), and nervonic (C24:1) acid and are not reported in Tables 2 or 3. The most abundant FA was oleic (C18:1) acid, followed by palmitic (C16:0), and stearic (C18:0) acids. Over three-quarters of the total fat content found consisted of these three FAs.

**Table 2:** Least squares means and standard errors of means for fatty acids of back fat from dairy steers grazed on winter rye and winter wheat.

Fatty acid	Cover crop			
	Winter rye		Winter wheat	
	Mean	SE	Mean	SE
	*% weight of total fat*			
C4:0, butyric	0.004^a^	0.001	0.001^b^	0.001
C14:0, myristic	3.43	0.099	3.32	0.103
C14:1*trans*, tetradecenoic	0.002^b^	0.000	0.005^a^	0.001
C14:1, myristoleic	1.32^b^	0.107	1.68^a^	0.111
C15:0, pentadecanoic	0.552	0.024	0.521	0.025
C16:0, palmitic	25.7	0.361	25.2	0.375
C16:1*trans*, hexadecenoic	0.310^a^	0.019	0.157^b^	0.020
C16:1, palmitoleic	5.66	0.274	6.04	0.285
C17:0, margaric	0.936	0.037	0.850	0.038
C17:1, margaroleic	0.001^a^	0.000	0.000^b^	0.000
C18:0, stearic	13.1	0.576	12.1	0.600
C18:1*trans*, elaidic	2.76	0.168	2.65	0.175
C18:1, oleic	40.3	0.620	41.4	0.646
C18:2*trans*, octadecadienoic	1.16^b^	0.037	1.31^a^	0.039
C18:2, conjugated linoleic	0.576	0.024	0.606	0.025
C18:2n-6, linoleic	2.83	0.099	2.80	0.104
C18:3n-6, γ-linolenic	0.021^b^	0.001	0.029^a^	0.001
C18:3n-3, α-linolenic	0.440	0.017	0.468	0.018
C20:0, arachidic	0.122	0.007	0.107	0.007
C20:1, gadoleic	0.166	0.013	0.155	0.014
C20:2n-6, eicosadienoic	0.051	0.002	0.053	0.002
C20:3, γ-eicosatrienoic	0.081	0.006	0.082	0.006
C20:3n-3, eicosatrienoic	0.063^a^	0.004	0.042^b^	0.004
C20:4n-6, arachidonic	0.035^b^	0.003	0.051^a^	0.003
C20:5n-3, eicosapentaenoic	0.010	0.001	0.009	0.001
C21:0, heneicosanoic	0.028^a^	0.002	0.020^b^	0.002
C22:2n-6, docosadienoic	0.006^a^	0.001	0.003^b^	0.001
C22:4n-6, docosatetraenoic	0.030	0.004	0.028	0.004
C22:5, docosapentaenoic	0.065	0.004	0.059	0.005
C22:6n-3, docosahexaenoic	0.003	0.001	0.002	0.001
C23:0, tricosanoic	0.013	0.001	0.013	0.001
	*% weight in fat sample*			
Saturated fat	44.3	0.830	42.6	0.864
*cis*-monounsaturated	47.7	0.907	49.6	0.944
*cis*-polyunsaturated	3.67	0.110	3.65	0.114
*trans* fat	4.26	0.186	4.16	0.194
Omega-3 fat	0.535	0.018	0.562	0.018
Omega-6 fat	3.04	0.099	3.02	0.103
Omega-6/3 ratio	5.76	0.192	5.41	0.199

^a,b^ Means within a row without common superscript letters are different at *P* < 0.05.

**Table 3:** Least squares means and standard errors of means for fatty acids of back fat for HOL, MVH crossbred, and NJV crossbred dairy steers.

Fatty acid	Breed group^1^						HOL vs crossbred
	HOL		MVH		NJV		
	Mean	SE	Mean	SE	Mean	SE	*P-value*
	*% weight of total fat*						
C4:0, butyric	0.004	0.001	0.002	0.001	0.003	0.001	0.42
C14:0, myristic	3.21^b^	0.121	3.28^a^^b^	0.121	3.63^a^	0.129	0.12
C14:1*trans*, tetradecenoic	0.003^b^	0.001	0.003^b^	0.001	0.005^a^	0.001	0.14
C14:1, myristoleic	1.37^b^	0.131	1.23^b^	0.131	1.89^a^	0.139	0.24
C15:0, pentadecanoic	0.516	0.029	0.532	0.029	0.561	0.031	0.40
C16:0, palmitic	25.6	0.442	24.8	0.442	26.0	0.468	0.71
C16:1*trans*, hexadecenoic	0.222	0.024	0.253	0.024	0.226	0.025	0.57
C16:1, palmitoleic	5.53	0.336	5.79	0.336	6.22	0.356	0.27
C17:0, margaric	0.943	0.045	0.867	0.045	0.869	0.048	0.19
C17:1, margaroleic	0.001	0.000	0.000	0.000	0.001	0.000	0.78
C18:0, stearic	13.2	0.705	12.8	0.705	11.9	0.748	0.35
C18:1*trans*, elaidic	3.12^a^	0.206	2.56^a^^b^	0.206	2.45^b^	0.218	0.02
C18:1, oleic	40.3	0.760	41.8	0.760	40.4	0.806	0.40
C18:2*trans*, octadecadienoic	1.23	0.046	1.25	0.046	1.22	0.049	1.00
C18:2, conjugated linoleic	0.616	0.029	0.580	0.029	0.577	0.031	0.31
C18:2n-6, linoleic	2.91	0.122	2.82	0.122	2.72	0.129	0.37
C18:3n-6, γ-linolenic	0.024	0.001	0.025	0.001	0.025	0.001	0.42
C18:3n-3, α-linolenic	0.428	0.021	0.476	0.021	0.459	0.023	0.14
C20:0, arachidic	0.118	0.008	0.117	0.008	0.109	0.009	0.59
C20:1, gadoleic	0.128^b^	0.016	0.202^a^	0.016	0.151^b^	0.017	0.02
C20:2n-6, eicosadienoic	0.052	0.002	0.053	0.002	0.051	0.003	0.81
C20:3, γ-eicosatrienoic	0.067^b^	0.007	0.099^a^	0.007	0.079^a^^b^	0.008	0.03
C20:3n-3, eicosatrienoic	0.052	0.005	0.058	0.005	0.048	0.005	0.95
C20:4n-6, arachidonic	0.038	0.004	0.047	0.004	0.044	0.004	0.13
C20:5n-3, eicosapentaenoic	0.008^b^	0.001	0.011^a^	0.001	0.011^a^	0.001	0.01
C21:0, heneicosanoic	0.025	0.002	0.023	0.002	0.024	0.002	0.46
C22:2n-6, docosadienoic	0.004	0.001	0.004	0.001	0.005	0.001	0.26
C22:4n-6, docosatetraenoic	0.025^b^	0.004	0.039^a^	0.004	0.024^b^	0.005	0.22
C22:5, docosapentaenoic	0.055^b^	0.005	0.072^a^	0.005	0.060^a^^b^	0.006	0.11
C22:6n-3, docosahexaenoic	0.002	0.001	0.003	0.001	0.002	0.001	0.21
C23:0, tricosanoic	0.013^a^^b^	0.001	0.014^a^	0.001	0.011^b^	0.001	0.43
	*% weight in fat sample*						
Saturated fat	44.0	1.017	42.8	1.017	43.5	1.078	0.50
*cis*-monounsaturated	47.7	1.111	49.3	1.111	49.1	1.178	0.28
*cis*-polyunsaturated	3.69	0.135	3.74	0.135	3.56	0.143	0.81
*trans* fat	4.61	0.228	4.09	0.228	3.93	0.242	0.04
Omega-3 fat	0.504^b^	0.022	0.589^a^	0.022	0.551^a^^b^	0.023	0.02
Omega-6 fat	3.10	0.122	3.06	0.122	2.93	0.129	0.48
Omega-6/3 ratio	6.18^a^	0.235	5.27^b^	0.235	5.32^b^	0.249	0.01

^a,b^ Means within a row without common superscript letters are different at *P* < 0.05.

^1^ HOL = Holstein; MVH = crossbreed comprised of Montbéliarde, Viking red, and Holstein; NJV = crossbreed comprised of Normande, Jersey, and Viking Red.

Fatty acids from steers grazed on WR and WW (Table 2) differed (*P* < 0.05) for butyric (C4:0), tetradecenoic (C14:1*trans*), myristoleic (C14:1), hexadecenoic (C16:1*trans*), margaroleic (C17:1), octadecadienoic (C18:2*trans*), γ-linolenic (C18:3n-6), eicosatrienoic (C20:3n-3), arachidonic (C20:4n-6), heneicosanoic (C21:0), and docosadienoic (C22:2n-6) acids. The sum of SFAs, *cis*-monounsaturated FAs, PUFAs, and *trans* fats were similar (*P* > 0.05) between steers grazed on WR and WW. Furthermore, n-3 FAs, n-6 FAs, and n-6/3 ratios were similar (*P* > 0.05) between steers grazed on WR and WW.

Differences in individual long-chain FAs (C20 to C22) from steers grazed on WR and WW may have been influenced by the different FA content in forages [34]. The amount of total fat in the diet may also influence the FA content in adipose tissue of steers. Microorganisms in the rumen may differ based on feeding systems; however, it is likely that the FA content and forage quality of WR and WW pastures contributed to the back fat FA content of steers based on the different FA concentrations among forage species [35].

Fatty acids from HOL, MVH, and NJV steers (Table 3) differed (*P* < 0.05) for myristic (C14:0), tetradecenoic (C14:1*trans*), myristoleic (C14:1), elaidic (C18:1*trans*), gadoleic (C20:1), γ-eicosatrienoic (C20:3), eicosapentaenoic (EPA; C20:5n-3), docosatetraenoic (22:4n-6), docosapentaenoic (22:5), and tricosanoic (C23:0) acids. Furthermore, elaidic (C18:1*trans*), tricosanoic (C23:0), eicosapentaenoic (EPA; C20:5n-3), and gadoleic (C20:1) acids were greater (*P* < 0.05) in crossbred steers compared to HOL steers. No differences were found for sums of saturated, *cis*-monounsaturated, *cis*-polyunsaturated, and n-6 FAs between HOL, MVH, and NJV steers. For *trans* fats, the HOL, MVH, and NJV steers were similar; however, HOL steers had greater (*P* < 0.05) *trans* fat than crossbred steers.

The MVH (0.589%) steers had greater (*P* < 0.05) n-3 FAs than HOL (0.504%) steers, and the HOL and MVH steers had similar (*P* > 0.05) n-3 FAs compared to NJV (0.551%) steers. The crossbred steers had greater (*P* < 0.05) n-3 FAs compared to the HOL steers. The greater concentration of long-chain PUFAs in the crossbred steers may have influenced the darker L* score observed in the steaks from crossbred steers (Table 1) due to lipid oxidation. Furthermore, the n-6/3 ratio was greater (*P* < 0.05) for HOL (6.18) steers compared to MVH (5.27) and NJV (5.32) steers. Subsequently, the HOL steers had a greater (*P* < 0.05) n-6/3 ratio compared to crossbred steers. These findings contradict those found in another study [11], which reported a greater n-6/3 ratio in Simmental (similar to Montbéliarde) bulls than HOL bulls. The genetics of the specific crossbreeds in the current study may have influenced the differences in FAs.

### Amino acid profiles

Least squares means and standard errors for AAs in steak from steers grazed on WR and WW, and for HOL, MVH, and NJV steer breed groups are in Table 4. The total protein (percent weight of meat sample) was similar for steers grazed on WR (10.3%) and WW (11.7%) (not reported in Table 4). Similarly, another study [36] reported that steers finished on mixed pasture, alfalfa, and pearl millet had similar total protein content in steak. For essential AAs, the steers grazed on WR (1.8, 1.7, 1.0, 0.98, 0.93, and 0.81) had greater (*P* < 0.05) percentages of lysine, leucine, valine, isoleucine, threonine, and phenylalanine than steers grazed on WW (1.7, 1.5, 0.96, 0.90, 0.85, and 0.76), respectively. However, histidine, methionine, and tryptophan were similar between steers grazed on WR and WW. A study conducted in Sweden [14] reported an increase in essential AA concentrations in steak from cows grazed on pasture compared to cows in a conventional system. For non-essential AAs, glutamine, aspartic acid, arginine, and serine were greater (*P* < 0.05) for steers grazed on WR compared to steers grazed on WW. Taurine was greater (*P* < 0.05) for steers grazed on WW (0.011%) compared to steers grazed on WR (0.005%), however taurine was the least concentrated AA found in the steak.

**Table 4:** Least squares means and standard errors for amino acids of meat from steers grazed on winter rye and winter wheat cover crops and for HOL, MVH crossbred, and NJV crossbred dairy steers.

Amino acid	Cover crop				Breed group^1^						HOL vs crossbred
	Winter rye		Winter wheat		HOL		MVH		NJV		
	Mean	SE	Mean	SE	Mean	SE	Mean	SE	Mean	SE	*P-value*
Essential amino acid	*% weight of total protein*										
Lysine	1.8^a^	0.05	1.7^b^	0.05	1.8	0.06	1.8	0.06	1.7	0.06	0.98
Leucine	1.7^a^	0.04	1.5^b^	0.04	1.6	0.05	1.6	0.05	1.6	0.05	0.72
Valine	1.0^a^	0.02	0.96^b^	0.02	0.99	0.03	1.0	0.03	0.98	0.03	0.77
Isoleucine	0.98^a^	0.02	0.90^b^	0.02	0.93	0.03	0.97	0.03	0.92	0.03	0.76
Threonine	0.93^a^	0.02	0.85^b^	0.02	0.89	0.03	0.92	0.03	0.87	0.03	0.86
Phenylalanine	0.81^a^	0.02	0.76^b^	0.02	0.78	0.02	0.80	0.02	0.78	0.02	0.74
Histidine	0.77	0.02	0.75	0.02	0.74	0.03	0.79	0.03	0.74	0.03	0.56
Methionine	0.52	0.02	0.49	0.02	0.50	0.02	0.52	0.02	0.50	0.02	0.64
Tryptophan	0.24	0.01	0.22	0.01	0.23	0.01	0.23	0.01	0.22	0.01	0.43
Non-essential amino acid											
Glutamine	3.1^a^	0.08	2.7^b^	0.09	2.9	0.10	3.0	0.10	2.8	0.11	0.91
Aspartic acid	1.9^a^	0.04	1.7^b^	0.05	1.8	0.05	1.8	0.05	1.8	0.06	0.96
Arginine	1.2^a^	0.03	1.1^b^	0.03	1.1	0.03	1.2	0.03	1.1	0.04	0.63
Tyrosine	1.2	0.04	1.1	0.04	1.1	0.05	1.2	0.05	1.2	0.05	0.61
Alanine	1.2	0.03	1.1	0.03	1.1	0.04	1.2	0.04	1.2	0.04	0.71
Glycine	0.93	0.03	0.87	0.03	0.88	0.04	0.90	0.04	0.92	0.04	0.65
Serine	0.74^a^	0.02	0.68^b^	0.02	0.71	0.02	0.73	0.02	0.70	0.02	0.78
Cysteine	0.21	0.01	0.20	0.01	0.20	0.01	0.21	0.01	0.20	0.01	0.53
Taurine	0.005^b^	0.001	0.011^a^	0.001	0.01	0.001	0.01	0.001	0.01	0.001	0.12

^a,b^ Means within a row for cover crops or dairy steers without common superscript letters are different at P < 0.05.

^1^ HOL = Holstein; MVH = crossbreed comprised of Montbéliarde, Viking red, and Holstein; NJV = crossbreed comprised of Normande, Jersey, and Viking Red.

The total protein content was similar for HOL (9.6%), MVH (11.7%), and NJV (11.7%) steers (not reported in Table 4). These results are similar to other studies [28,37], which reported similar total protein concentrations in beef from dairy steers of different breeds. No differences in essential and non-essential AAs were found between HOL and crossbred steers.

### Consumer sensory evaluation of beef

Least squares means and standard error of means for sensory attributes are in Table 5. For overall consumer liking, means for WW (72.0) steaks were greater (*P* < 0.05) than means for WR (66.7) steaks. For flavor liking, texture liking, and juiciness, means for WW steaks were greater (*P* < 0.05) than WR steaks. Furthermore, the means for WR steaks were greater (*P* <0.05) for toughness and off-flavor than WW steaks. In another study [31], which compared sensory attributes of steaks from steers grazed on mixed pasture, alfalfa, and pearl millet, steaks from steers finished on pearl millet had lower off-flavor than steers finished on mixed pasture and alfalfa. Bjorklund et al. [6] reported that consumers preferred steaks from conventionally raised steers over steaks from grass-fed steers. However, some consumers in that study did prefer the grass-fed steaks indicating there is market potential for organic grass-fed beef.

**Table 5:** Least squares means and standard errors for sensory attributes of steaks for steers grazed on winter rye and winter wheat and for HOL, MVH crossbred, and NJV crossbred dairy steers.

Sensory attribute	Cover crop			Breed group^1^				HOL vs crossbred
	Winter rye	Winter wheat	SE^4^	HOL	MVH	NJV	SE^4^	*P-value*
Overall^2^	66.7^b^	72.0^a^	1.4	67.2^b^	69.2^a^^b^	71.8^a^	1.6	0.02
Flavor^2^	66.5^b^	70.3^a^	1.5	66.5^b^	67.9^a^^b^	70.7^a^	1.6	0.04
Texture^2^	66.1^b^	74.3^a^	1.4	67.5^b^	69.4^b^	73.8^a^	1.6	0.01
Toughness^3^	8.9^a^	7.3^b^	0.3	8.6^a^	8.4^a^	7.4^b^	0.3	0.03
Juiciness^3^	8.0^b^	9.2^a^	0.3	7.8^b^	9.2^a^	8.9^a^	0.4	<0.01
Off-flavor^3^	5.6^a^	4.8^b^	0.4	5.3	5.3	5.0	0.4	0.58

^a,b^ Means within a row for cover crops or dairy steers without a common letter are different at *P* < 0.05.

^1^ HOL = Holstein; MVH = crossbreed comprised of Montbéliarde, Viking red, and Holstein; NJV = crossbreed comprised of Normande, Jersey, and Viking Red.

^2^ Overall flavor and texture liking/disliking: 0 = greatest imaginable disliking; 120 = greatest imaginable liking.

^3^ Toughness, juiciness, and off-flavor: 0 = none; 20 = extremely tough, extremely juicy, or extremely intense.

^4^ Standard errors were the same for cover crops and breeds.

For breed groups, the NJV (71.8) steaks were greater (*P* < 0.05) for overall liking than HOL (67.2) steaks, but were similar to MVH (69.2) steaks. These results are similar to those found in Nuernberg et al. [11], which reported that HOL and Simmental (breed similar to Montbéliarde) steaks had similar overall liking. Furthermore, flavor likeness was greater (*P* < 0.05) for NJV (70.7) steaks compared to HOL (66.5) steaks, but was similar to MVH (67.9) steaks. For texture likeness, the NJV (73.8) steaks were greater (*P* < 0.05) than both HOL (67.5) and MVH (69.4) steaks. The NJV (7.4) steaks were lower (*P* < 0.05) for toughness intensity than both HOL (8.6) and MVH (8.4) steaks. For juiciness intensity, both NJV (8.9) and MVH (9.2) steaks were greater (*P* < 0.05) than HOL (7.8) steaks. No differences were found for off-flavor between breeds. The crossbred steaks had greater (*P* < 0.05) overall, flavor, and texture liking compared to HOL steaks. For the intensity of sensory attributes, crossbred steaks had greater (*P* < 0.05) juiciness intensity and less (*P* < 0.05) toughness intensity than HOL steaks. In another study [38], Brown Swiss (similar ancestry to Montbéliarde) steaks had lower toughness than HOL steaks; however, overall sensory attributes and juiciness were not influenced by breed. Specific crossbreeds in an organic system may have influenced sensory attribute differences in the current study.

Percentages of like/dislike categories for WR and WW steaks, and HOL, MVH, and NJV steaks are in Table 6. According to the likeness scale, more consumers (*P* < 0.05) slightly liked steak from WW (76.5%) than WR (63.6%) steers, and more (*P* < 0.05) consumers moderately liked steak from WW (34.0%) than WR (23.5%) steers. A similar (*P* > 0.05) proportion of consumers liked the steak very much and extremely liked steak from WR and WW steers.

**Table 6:** Means for overall like/dislike categories for steers grazed on winter rye and winter wheat and for HOL, MVH crossbred, and NJV crossbred dairy steers.

Sensory attribute^2^	Cover crop		Breed group^1^			HOL vs crossbred
	Winter rye	Winter wheat	HOL	MVH	NJV	*P-value*
Like slightly, 60 to 120, %	63.6^b^	76.5^a^	62.0^b^	70.8^a^	77.3^a^	<0.01
Like moderately, 81 to 120, %	23.5^b^	34.0^a^	24.5	30.6	31.0	0.05
Like very much, 93 to 120, %	8.6	11.4	9.7	11.6	8.8	0.83
Like extremely, 104 to 120, %	3.1	1.5	2.8	1.9	2.3	0.54

^a,b^ Means from chi-squared test within a row for cover crops or dairy steers without a common letter are different at P < 0.05.

^1^ HOL = Holstein; NJV = crossbreed comprised of Normande, Jersey, and Viking red; MVH = crossbreed comprised of Montbéliarde, Viking red, and Holstein.

^2^ Overall liking/disliking: 0 = greatest imaginable disliking; 120 = greatest imaginable liking.

Furthermore, more (*P* < 0.05) consumers slightly liked NJV (77.3%) and MVH (70.8%) steaks than HOL (62.0%) steaks. To complement this, more (*P* < 0.01) consumers slightly liked crossbred steak than HOL steak. Consumers who moderately liked steaks were similar (*P* > 0.05) for HOL, MVH, and NJV steers; however, more (*P* < 0.05) consumers moderately liked crossbred steak than HOL steak. A similar (*P* > 0.05) proportion of consumers liked steak very much and extremely liked steak from HOL, MVH, and NJV steers, and from HOL and crossbred steers, respectively.

The likeness of steak results indicates that the magnitude of differences between the WR and WW, and the HOL, MVH, and NJV steers found in the sensory study only influenced consumers to slightly like or moderately like the WW more than the WR steaks and the crossbred more than the HOL steaks. In total, only 10.0% of consumers liked the steaks very much and only 2.3% of consumers extremely liked the steaks, indicating that sensory attribute results found in this study shows that differences between forages and breeds only have a slight or moderate effect on the actual sensory attributes on the resulting beef product.

### Principal component analysis and correlation

Correlation coefficients between selected meat quality variables are in Table 7. For objective color scores, only a* and b* were correlated with each other (0.94). The WBSF was negatively correlated with flavor simply because of the positive relationship of tenderness and flavor in beef [39]. There were no significant associations with WBSF and consumer acceptability scores. However, the correlation of WBSF and overall liking was greater in this study than reported by Destefanis et al. [25], (-0.61 (current study) versus -.0.37). The correlations in the current study may not be significant between some variables because of the smaller sample size as compared to other studies [25]. Influences of breed on meat quality characteristics may affect correlations between meat quality and sensory data. Gagaoua et al [39] and Cuvelier et al. [40] reported that animal characteristic, including breed must be taken into account when determining relationships between sensory data and meat quality. Differences in breeds across studies may affect the results of specific combinations of meat quality and sensory characteristics.

**Table 7:** Correlation of coefficients between select meat quality variables.

	L*^1^	A*^1^	B*^1^	WBSF^2^	Overall	Flavor	Texture	Toughness	Juiciness
L*									
A*	0.41								
B*	0.57	0.94**							
WBSF	0.30	-0.19	0.01						
Overall liking	-0.43	-0.47	-0.56	-0.61					
Flavor	-0.38	-0.21	-0.38	-0.85**	0.93**				
Texture	-0.41	-0.52	-0.61	-0.64	0.99**	0.93**			
Toughness	0.30	0.54	0.59	0.47	-0.98**	-0.86*	-0.97**		
Juiciness	-0.61	-0.31	-0.37	-0.36	0.82*	0.68	0.74	-0.78	
Off-flavor	0.32	0.51	0.51	0.58	-0.95**	-0.86**	-0.96**	0.95**	-0.72

^1^L* = brightness (0 = black; 100 = white); a* = redness/greenness (positive values = red; negative values = green); b* = yellowness/blueness (positive values = yellow; negative values = blue).

^2^ Warner-Bratzler shear force

** = *P* < 0.01;

* = *P* < 0.05, levels of significance

The results of the principal component analysis are in Table 8. For the 5 principal components. The analysis shows that 66% of the total variation is explained by the first principal component, 84.6% by the first two principal components, and 93.5% by the first three principal components. Specifically, 93.5% of the total variance in the 10 considered variables can be reduced into three new variables.

**Table 8:** Results from the principal component analysis for the first 5 principal components.

Component	Eigenvalues	% of variance	Cumulative variance, %
1	6.6	66.2	66.2
2	1.8	18.4	84.6
3	0.9	8.9	93.5
4	0.5	5.5	99.0
5	0.1	1.0	100.0

**Fig 1:**
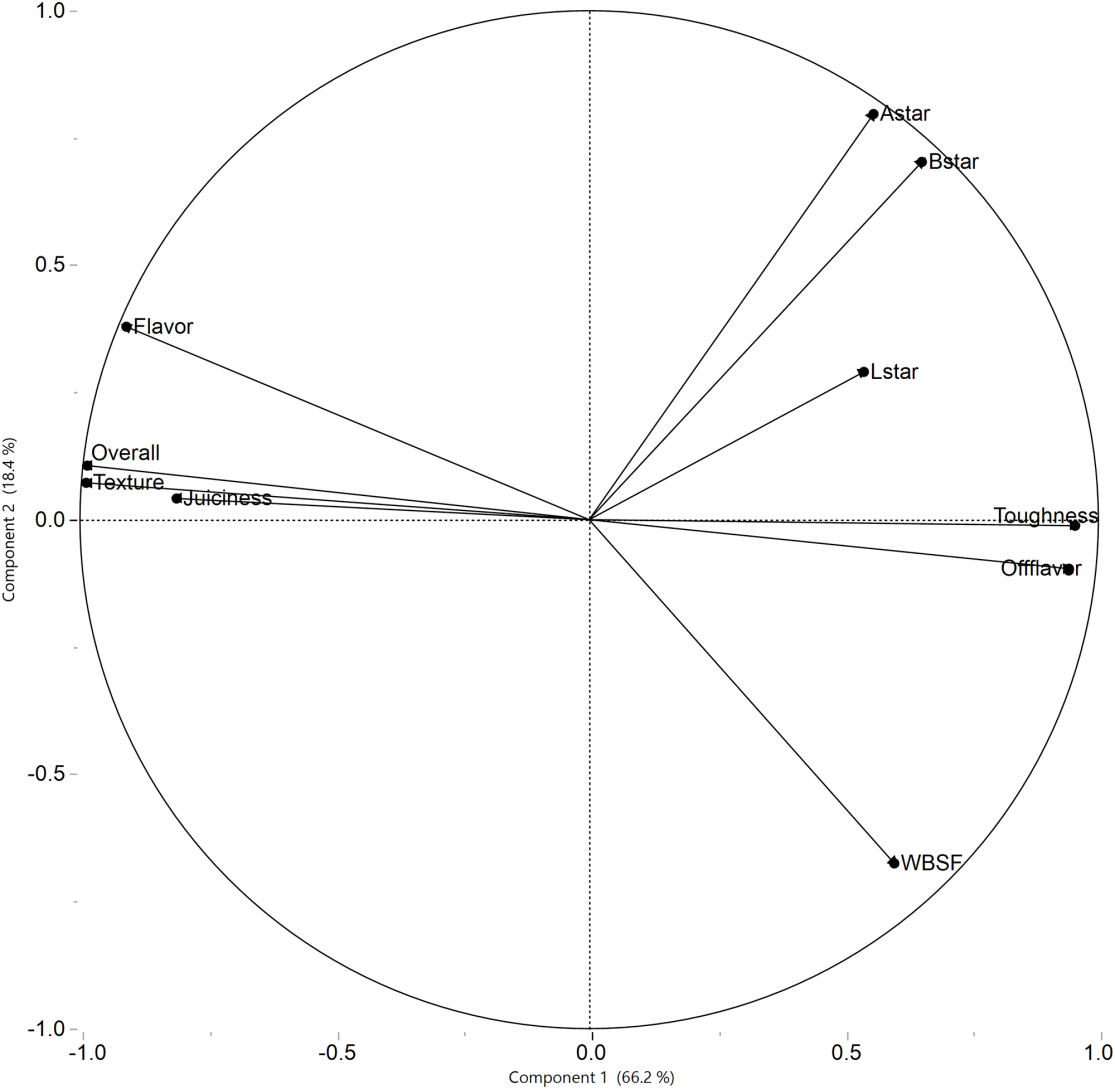
Loading plot for the first two components from principal component analysis.

In the loading plot from the principal component analysis (Fig 1), the variables on the right side in the loading plot are related to toughness of steaks and are positively correlated, and represent the first principal component which includes L*, a*, b*, toughness, off flavor and WBSF. The variables on the left of the loading plot (flavor, overall, texture, juiciness) are positively correlated and represent the second principal component. Overall, principal component analysis showed the meat quality variables that have relationships among the original 10 variables chosen for analysis.

## Conclusions

Organic bull calves may add value and economic diversity for organic dairy producers if utilized for organic meat products. This study examined the potential for an organic, forage-based diet, including winter wheat and winter rye grazed for 7 weeks in the spring, to supply adequate nutrition for marketable meat quality of dairy steers. Increased forage in the rations of dairy cattle has been reported to improve the FA profile of dairy and beef products. In our study, the FAs from crossbred steers consisted of a greater n-3 FA concentration compared to purebred HOL steers. Furthermore, a lower n-6/3 FA ratio was found in crossbred compared to HOL steers. In sensory evaluation panels, consumers liked steak from crossbred steers more than HOL steaks, and steak from steers grazed on WW over WR. Steak from crossbred steers rated higher than HOL steaks in overall, flavor, and texture likeness. Toughness and juiciness intensities were rated lower and higher, respectively, for crossbred over HOL steaks. Improvements in the nutritional quality of beef may have the potential to improve consumer acceptability of beef and human health.
